# Evolutionary plasticity of zoonotic porcine Deltacoronavirus (PDCoV): genetic characteristics and geographic distribution

**DOI:** 10.1186/s12917-022-03554-4

**Published:** 2022-12-22

**Authors:** Amina Nawal Bahoussi, Pei-Hua Wang, Pir Tariq Shah, Hongli Bu, Changxin Wu, Li Xing

**Affiliations:** 1 Institutes of Biomedical Sciences, Shanxi University, 92 Wucheng Road, Taiyuan, 030006 Shanxi province China; 2 Department of Laboratory Medicine, The Fourth People’s Hospital of Taiyuan, 231 Xikuang St, Taiyuan, 030053 Shanxi province China; 3 Shanxi Provincial Key Laboratory of Medical Molecular Cell Biology, Shanxi University, 92 Wucheng Road, Taiyuan, 030006 China; 4 Shanxi Provincial Key Laboratory for Prevention and Treatment of Major Infectious Diseases, 92 Wucheng Road, Taiyuan, 030006 China; 5 The Key Laboratory of Chemical Biology and Molecular Engineering of Ministry of Education, Shanxi University, Taiyuan, 030006 China

**Keywords:** Porcine deltacoronavirus (PDCoV), Zoonotic pathogen, Coronavirus, Recombination, Linear B cell epitope, Spike glycoprotein

## Abstract

The emergence and rapid spread of the acute respiratory syndrome coronavirus-2 have confirmed that animal coronaviruses represent a potential zoonotic source. Porcine deltacoronavirus is a worldwide evolving enteropathogen of swine, detected first in Hong Kong, China, before its global identification. Following the recent detection of PDCoV in humans, we attempted in this report to re-examine the status of PDCoV phylogenetic classification and evolutionary characteristics. A dataset of 166 complete PDCoV genomes was analyzed using the Maximum Likelihood method in IQ-TREE with the best-fitting model GTR + F + I + G4, revealing two major genogroups (GI and GII), with further seven and two sub-genogroups, (GI a-g) and (GII a-b), respectively. PDCoV strains collected in China exhibited the broadest genetic diversity, distributed in all subgenotypes. Thirty-one potential natural recombination events were identified, 19 of which occurred between China strains, and seven involved at least one China strain as a parental sequence. Importantly, we identified a human Haiti PDCoV strain as recombinant, alarming a possible future spillover that could become a critical threat to human health. The similarity and recombination analysis showed that PDCoV spike ORF is highly variable compared to ORFs encoding other structural proteins. Prediction of linear B cell epitopes of the spike glycoprotein and the 3D structural mapping of amino acid variations of two representative strains of GI and GII showed that the receptor-binding domain (RBD) of spike glycoprotein underwent a significant antigenic drift, suggesting its contribution in the genetic diversity and the wider spread of PDCoV.

**Supplementary Information:**

The online version contains supplementary material available at 10.1186/s12917-022-03554-4.

## Introduction

Anthropogenic climate change and the industrial revolution are known to increase the risk of pathogen spillover from host reservoirs, leading to the emergence of new or unexpected infectious diseases [1, 2]. Zoonotic transmission of viruses from nonhuman animals is driving most emerging human infectious diseases and negatively impacting public health. After several outbreaks throughout history, the devastating socioeconomic impact of the recent Severe Acute Respiratory Syndrome Coronavirus 2 pandemic (SARS-CoV-2) makes the coronaviruses (CoVs) an urgent global threat and a high-profile example of a highly pathogenic zoonotic virus [3, 4]. Coronaviruses, classified into alpha, beta, gamma, and delta genera (α-CoV, β-CoV, γ-CoV, δ-CoV) in the *Coronaviridae* family of the order *Nidovirales,* are increasing attention and given research priority [5]. The recent detection of porcine delta coronavirus (PDCoV) and feline–canine recombinant Alpha-genus CoV in human patients demonstrates that many more animals may serve as either reservoirs or intermediate hosts [6]. Therefore, we find it of utmost importance to understand the PDCoV spread and help explicitly address its concurrent changes to prevent future outbreaks.

*Deltacoronavirus* genus has the smallest genome among all coronaviruses, defined using the complete genome sequencing and comparative genome analysis of mammalian and avian strains [7]. Deltacoronavirus in pigs was first detected in 2012 in Hong Kong, China, and named PDCoV HKU-15 prototype [7]. Two years later, in February 2014, PDCoV was identified as the etiological agent of the pig diarrheic outbreaks that started first in Ohio, USA, before spreading globally [8–10]. PDCoV was reported in many countries of the Americas and Asia, including Canada [11], Peru [12], Mexico [13], Thailand [14], Vietnam [15], Laos [16], South Korea [17], and Japan [18]. Clinically, PDCoV is indiscernible from porcine epidemic diarrhea viruses (PEDV), Transmissible Gastroenteritis Coronaviruses (TGEV), or porcine rotavirus [19], causing diarrhea, vomiting, dehydration, lethargy, and histopathological damages such as acute necrosis of intestinal epithelial cells, intestinal villi contraction and shedding, intestinal wall thinning [19, 20] with a mortality rate of ~ 30–40% among infected piglets [21]. Contrary to PEDV and TGEV, PDCoV has demonstrated an extensive host range, capable of naturally infecting diverse species, including all kinds of pigs [22], calves [23], chickens, and poultry [24], in addition to human-derived cell lines [25, 26]. Recently, a natural zoonotic spillover of PDCoV has been reported in rural and sub-rural areas in the Republic of Haiti, an island nation in the northern Caribbean Sea [27]. Three PDCoV strains: Haiti/human/0081–4/2014 (GenBank ID: MW685622), Haiti/human/ 0329–4/2015 (GenBank ID: MW685624), and Haiti/human/ 0256–1/2015 (GenBank ID: MW685623) were identified in the plasma samples of three Haitian children with acute undifferentiated febrile illness between 2014 and 2015 [27]. The Republic of Haiti repopulated the swine herds from North America, Europe, and China after local swine herds were eradicated in 1978 due to African Swine Fever (ASF) infection [28]. The molecular clock calibration inferred that PDCoVs had been circulating in Haitian pigs for a few years before infecting human patients [27].

PDCoV is an enveloped virus comprising a single-stranded positive-sense RNA genome of approximately 25.4 kb and nine open reading frames (ORFs) [29, 30]. The three-quarters of the genome at the 5′ terminus are occupied by ORF1a/b, encoding the polyprotein precursors (pp1a and pp1b) cleaved by papain-like protease (PL-pro) and serine-type 3C-like proteases (3CLpro) to generate 15 mature nonstructural proteins (nsps), responsible for PDCoV replication [29]. The quarter of the genome at the 3′ terminus encodes four structural proteins, including the spike glycoprotein (S), the envelope protein (E), the membrane protein (M), and the nucleocapsid protein (N), in addition to three accessory proteins NS6, NS7 and NS7a [29]. PDCoV has been demonstrated to develop evolving strategies and escape the innate antiviral immune response of the host. The nonstructural protein Nsp5 of PDCoV cleaves the NF-κB essential modulator (NEMO) or STAT2 in the JAK-STAT pathway through its protease activity to inhibit the production of interferon (INF) [31, 32]. Similarly to other coronavirus invasion mechanisms, Nsp5 of PDCoV cleaves the porcine mRNA-decapping enzyme 1a (pDCP1A) to prevent its antiviral activity [33]. Meanwhile, Nsp10 and Nsp15 inhibit IFN-β production by compromising the activation of IRF3 and NF-κB transcription factors [34, 35]. The NS6, an important virulence factor of PDCoV [36], interacts with RIG-I/MDA5 to inhibit the production of IFN-β [37], and the NS7a inhibits IFN-β production by disrupting the interaction of I-kappa B kinase ε (IKKε) with the transcription factors TRAF3 and IRF3 [38].

The spike glycoprotein of coronaviruses is the main structural protein of the viral envelope, essential in binding specific surface receptors and mediating entry into the host cells. The spike glycoprotein consists of an amino-terminal trimeric S1 subunit used for receptor binding and an S2 subunit of carboxyl-terminus responsible for the host and viral membrane fusion [39, 40]. Substitution of the PDCoV S glycoprotein or its receptor-binding domain (RBD) by the S glycoprotein of sparrow deltacoronavirus (SpDCoV) strain HKU17 or the RBD of ISU73347 SpDCoV strain attenuated the virulence and intestinal tropism [41]. The cellular aminopeptidase N (APN) seems to be a receptor that mediates the PDCoV entry into the host cell by binding to the RBD of the S1 subunit [26, 42, 43]; however, the use of APN-specific antibodies and inhibitors or APN knockdown could not completely inhibit PDCoV infection, suggesting other unknown molecules involved in PDCoV infection [41, 43, 44]. Recently, Sialic acid (SA) was identified as an attachment receptor facilitating virus infection [45]. The S glycoprotein is a major inducer of host humoral immunity and is frequently used in vaccine design. The antiserum specific for the C-terminal domain (CTD) of the S1 subunit of PDCoV S glycoprotein has demonstrated the strongest PDCoV inhibitory effect, confirming that S1 subunit contains the main neutralizing epitopes [46]. Huang et al. used CRISPR/Cas9 gene editing and homologous recombination technologies to construct a recombinant pseudorabies virus rPRVXJdelgE/gI/TK-S expressing PDCoV S glycoprotein, which can induce high levels of antibodies in mice [47]. However, until now, no PDCoV vaccine has been commercially available.

China is a large pig-raising country with a vast territory and dense population. The high prevalence of PDCoV among pig herds in China poses a more obvious threat to the health of animals and human beings [48]. Since the first discovery in Hong Kong in 2012, PDCoV infection has been reported in 26 provinces in China, including Henan [49], Hebei [50], Guangdong [51], Guangxi [52], Shandong [53], and Sichuan [54]. Dong et al. indicated in a retrospective study that PDCoV was circulating in pig herds in China earlier than 2012 when two samples collected from Anhui were detected to be PDCoV positive [55]. Epidemiological studies revealed that PDCoV infection is prevalent in central and southern China, where pig production and transportation frequency are higher [56]. In southern China, PDCoV represents the second most prevalent virus in diarrhea pig herds, with a positive rate between 19.62 and 36.18%, during 2012–2018 [57, 58]. Therefore, the recent Haiti swine-to-human PDCoV transmission [27] and the lesson from the previous H1N1 outbreak by the swine-origin human influenza (S-OIV) [59] generated our interest in assessing the molecular characteristic and global distribution of the emerging PDCoVs with an emphasis on China strains to facilitate the surveillance and prevent the zoonotic spillover of this pathogen. We elucidated the evolutionary history of PDCoVs using the phylogenetic and recombination analysis of all publicly available complete genomic sequences isolated during the two past decades and provided valuable information for the public health prevention and control strategies.

## Materials and methods

### Dataset acquisition

All available PDCoV full-length genome nucleotide sequences collected between 2004 and 2020 from China (85), the United States (43), Thailand (14), South Korea (7), Japan (8), Vietnam (4), Haiti (3), Laos (1), and Peru (1), were retrieved from the NCBI GenBank database (http://www.ncbi.nlm.nih.gov/; accessed on May 2022) and analyzed in this report. Sequences with (a) 100% similarity, (b) truncated sequences, or (c) sequences with missing parts were discarded. A dataset of 166 PDCoVs with complete coding sequence information was involved in our final analysis.

### Phylogenetic analysis of PDCoVs

PDCoV sequence alignments were performed with ClustalW using the Molecular Evolutionary Genetics Analysis-11 (MEGA-11) [60]. A Maximum Likelihood (ML) phylogenetic tree of PDCoV full-length sequences was inferred with IQ-TREE multicore version 1.6.12, using the best-fitting model GTR + F + I + G4, and ML phylogenetic trees of the complete ORF1a/b sequences, complete ORF Spike sequences, and the complete sequences encoding E, M, and N proteins (ORFs E-N) were inferred using the best fitting models GTR + F + I + G4, TN + F + I + G4, and TNe + I + G4, respectively with 1000 bootstraps [61]. The resulting trees were visualized and modified using the FigTree v1.4 program (http://www.figtree.org/). The internal node numbers indicate the bootstrap values. Strains are formatted as GenBank accession number: virus name(country-year of collection).

### Similarity analysis of the full-length genome

The nucleotide sequence similarities between nine PDCoV representative viruses from China were determined using SimPlot ver. 3.5.1 software [62]. PDCoV AH2019-H (GenBank ID: MN520198.1) was used as a query strain.

### Amino acid variability analysis

The complete nucleotide coding sequences of structural proteins, including S, E, M and N of 166 PDCoV strains, were aligned with ClustalW using the MEGA-11 software, then translated and edited using the BioEdit version 7.2.5. The amino acid variability was determined using the PVS (Protein Variability Server) with the Wu-Kabat variability coefficient method [63]. The variability coefficient was calculated with the following formula: variability = N*k/n, where N represents the number of sequences in the alignment, k represents the number of different amino acids at a given position, and n is the time that the most commonly recognized amino acid at that position is available [64].

### Recombination screening

The occurrence of potential recombination events between full-length genome sequences of 166 strains in this study was analyzed using seven algorithms of the RDP4 software package, including RDP, GENECONV, Bootscan, MaxChi, Chimaera, SiScan, and 3Seq [65]. The recombination event identified by at least four of the seven methods was accepted in this report. Phylogenetic trees based on the indicated genomic regions of PDCoV strains involved in the recombination were generated using the Maximum Likelihood method in MEGA-11 software [66]. The nucleotide genomic sequences are relative to CHN-GX81–2018 (GenBank ID: MN173781.1) strain.

### Linear B cell epitopes prediction and three-dimensional visualization of PDCoV S protein

The PDCoV S glycoprotein Linear B cell epitopes were predicted using Bepipred-2.0 software, operating under IEDB (the immune epitope database, https://www.iedb.org/). The epitope and non-epitope amino acids were defined from the crystal structure by a Random Forest algorithm. Residues scores above the threshold (> 0.5) were predicted to be an epitope part [67]. The 3D structure of S1 subunits (aa 51–419) of the two PDCoV representative strains from GI and GII, [AH2019-H (GenBank ID: MN173781) and CHN-GX81–2018 (GenBank ID: MN520198.1) respectively], were modeled using the I-TASSER server (Iterative Thread ASSEmbly Refinement) [68]. I-TASSER first identified the structural templates from the RCSB protein data bank (PDB) using the multiple threading approach LOMETS. Subsequently, the full-length atomic models were generated using iterative template-based fragment assembly simulations.

## Results

### Phylogenetic tree reveals a great genetic diversity of China PDCoV strains

**Fig. 1:**
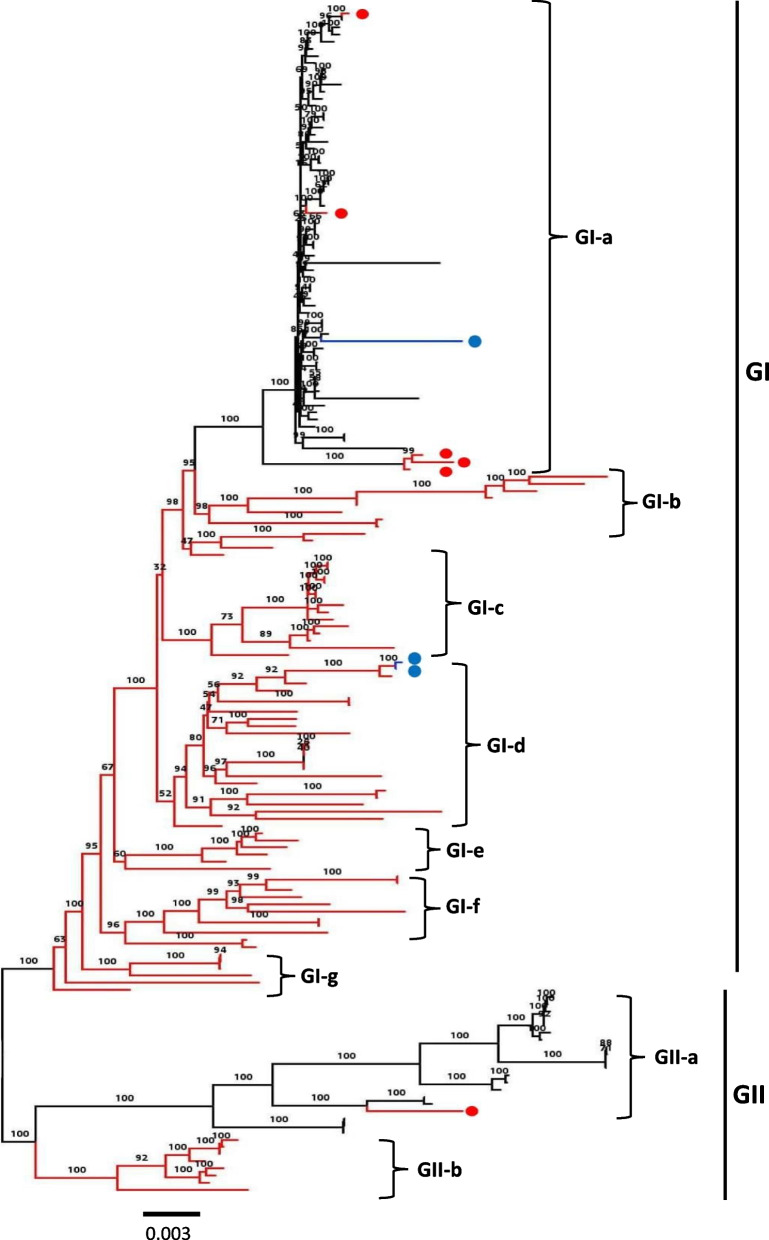
ML phylogenetic tree based on the full-length genome sequence  of 166 PDCoV strains was generated using IQ-TREE multicore version 1.6.12 with the best-fit model GTR + F + I + G4 with 1000 bootstraps. The China viruses are indicated with red branches. The China viruses that are sorted in  GI-a and GII-a subgenogroups are shown with red branches plus red circles. The human Haiti PDCoV strains are indicated with blue circles. The scale bar represents 0.003 nucleotide substitutions per site. The information on each virus is detailed in Supplementary Fig. 1

**Fig. 2:**
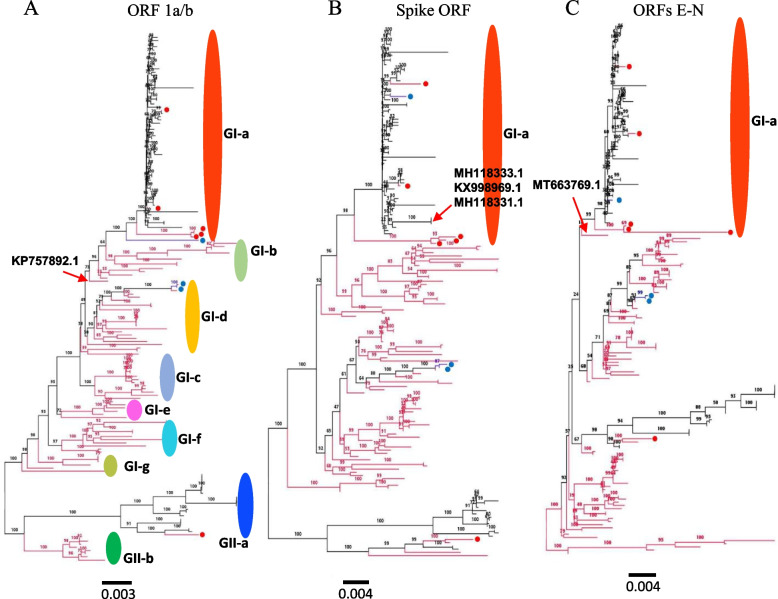
ML phylogenetic trees based on indicated genomic fragments of 166 PDCoV strains. (**A**) ORF 1a/b complete nucleotide sequences; (**B**) ORF Spike complete nucleotide sequences; (**C**) ORFs E-N complete nucleotide sequences. Multiple sequence alignment was performed using ClustalW. The phylogenetic trees were constructed in IQ-TREE multicore version 1.6.12 using the best-fitting model GTR + F + I + G4 for (**A**), TN + F + I + G4 for (**B**), and TNe + I + G4 for (**C**) with 1000 bootstraps. The China PDCoV viruses are indicated in red branches or red circles. The human Haiti PDCoV strains are indicated in blue circles. The scale bar represents nucleotide substitutions per site. The information on each virus in (**A**), (**B**), and (**C**) is detailed in Supplementary Figs. 2, 3, and 4, respectively

To review the genetic characteristics and infer the evolutionary history of the global PDCoV strains during the past two decades, we performed a phylogenetic analysis of 166 full-length viruses collected between 2004 and 2020 and distributed worldwide, 166 complete ORF1a/b coding sequences, 166 complete ORF spike region, and 166 complete ORFs E-N coding sequences, using the Maximum Likelihood method in IQ-TREE with the best-fitting models GTR + F + I + G4, GTR + F + I + G4, TN + F + I + G4, and TNe + I + G4, respectively [61]. As shown in Fig. 1 and Supplementary Fig. 1, the phylogenetic tree indicates that PDCoV strains cluster into two main genogroups: GI and GII. The GI is further subdivided into seven subgenogroups: GI (a, b, c, d, e, f and g) and the GII into two subgenogroups: GII (a and b). The GI-a consists mainly of isolates from the United States (2014/2015), Japan (2014), South Korea (2016), and Peru (2019) (Fig. 1, Table 1). Strains belonging to GI-b, GI-c, GI-e, GI-f, and GI-g are restricted to China (2004–2020). Viruses in the GII-a are mainly limited to South Asia, including Thailand (*n* = 14, 2013–2018), Vietnam (*n* = 4, 2015–2016), and Laos (*n* = 1, 2016), whereas GII-b is limited to strains collected in China in 2018, including AH2018–93 (GenBankID: MN520199.1), AH2018–81 (GenBankID: MN520193.1), AH2018–94 (GenBankID: MN520200.1), CHN-GX09–2018 (GenBankID: MN173782.1), CHN-GX01–2018(GenBankID: MK359104.1), CHN-GX12–2018(GenBankID: MN173780.1), CHN-GX11–2018(GenBankID: MN173779.1), and CHN-GX81–2018(GenBankID: MN173781.1)(Fig. 1, Supplementary Fig. 1). Importantly, PDCoV strains isolated from China are largely distributed and are identified in all subgenogroups, indicating the extensive genetic diversity of China strains (Fig. 1 and Table 1). The GI-b contains the earliest strain, CHN-AH-2004 (GenBank ID: KP757890.1), isolated in Anhui, China, in 2004. The GI-a and GII-a also contain strains from China: SD2019–426 (GenBank ID: MN520191.1), AH2019-H (GenBank ID: MN520198.1), CH-HLJ-20 (GenBank ID: MZ802955.1), CHzmd2019 (GenBank ID: MN781985.1), and CHN-GD16–05 (GenBank ID: KY363868.1), in GI-a, and CH/GX/1468B/2017 (GenBank ID: MN025260.1) in GII-a. Intriguingly, the Haitian human-infecting strains: PDCoV/Haiti/Human/0256–1/2015 (GenBank ID: MW685623.1) cluster into GI-a subgroup and PDCoV/Haiti/Human/0329–4/2015 (GenBank ID: MW685624. 1) together with PDCoV/Haiti/Human/0081–4/2014 (GenBank ID: MW685622.1) cluster into GI-d close to China strains (Supplementary Fig. 1). The phylogenetic tree based on the complete individual gene ORF1a/b revealed that PDCoV strains consistently cluster into two main genogroups (GI and GII) with seven and two further subgenogroups, respectively, as defined by results of the full-length genome-based genotyping classification (Fig. 2A, Supplementary Fig. 2) except for one virus CHN-JS-2014 (GenBank ID: KP757892.1) that shifted from GI-d in the full-length genome-based tree (Fig. 1 and Supplementary Fig. 1) to GI-b subgenogroup in the ORF 1a/b region-based tree (Fig. 2A and Supplementary Fig. 2), highly supporting the genotyping classification (Fig. 1). Phylogenetic tree topology based on the complete ORF Spike and ORFs E-N exhibited that PDCoV strains maintained one cluster of relative consistency, GI-a, contrary to the other strains (from China) that were phylogenetically incongruent (Fig. 2B, C, Supplementary Figs. 3, and 4). GI-a in ORF Spike-based tree (Fig. 2B and Supplementary Fig. 3) contains three more viruses (GenBank ID: MH118333.1, KX998969.1, and MH118331.1) that are sorted in GII-a subgenogroup of the full-length genome-based tree (Fig. 1 and Supplementary Fig. 1). GI-a in ORFs E-N-based tree (Fig. 2C and Supplementary Fig. 4) contains one more virus CHN-TS1–2019 (GenBank ID: MT663769.1) that clustered within GI-c subgenogroup of the full-length genome-based tree (Fig. 1 and Supplementary Fig. 1).

**Table 1:** Geographic distribution of PDCoV genogroups

	China	Other countries in Asia	America	Total
GI-a	China (5)	Japan (8), South Korea (7)	USA (43), Peru (1), Haiti (1)	65
GI-b	China (12)	/	/	12
GI-c	China (14)	/	/	14
GI-d	China (22)	/	Haiti (2)	24
GI-e	China (6)	/	/	6
GI-f	China (11)	/	/	11
GI-g	China (6)	/	/	6
GII-a	China (1)	Thailand (14), Vietnam (4), Laos (1)	/	20
GII-b	China (8)	/	/	8

**Fig. 3:**
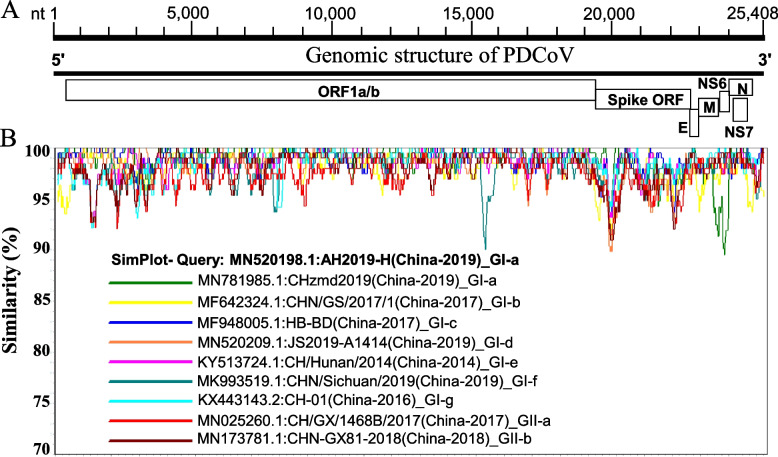
Similarity analysis of the nucleotide sequence of nine PDCoV full-length representative viruses. **a** Scheme of the ORF regions contained in the PDCoV full-length genome sequences: ORF1a/b, Spike ORF, envelope protein ORF, membrane protein ORF, NS6 ORF, nucleocapsid protein ORF and NS7 ORF from the 5′ terminus to the 3′ terminus of the genome. E, envelope protein ORF; M, membrane protein ORF; N, nucleocapsid protein ORF; NS6, NS6 protein ORF; NS7, NS7 protein ORF. **b** Similarity map of nine PDCoVs representing each subgenogroup with AH2019-H from GI-a (GenBank ID: MN520198.1) as a query strain using SimPlot ver. 3.5.1 software. Each different color line represents a PDCoV isolate. The horizontal axis represents the position of nucleotide sequences, and the vertical axis represents the nucleotide similarity percentage. The nucleotide position (nt) is relative to the strain CHN-GX81–2018 (GenBank ID: MN173781.1)

Since China PDCoV strains demonstrated the widest genetic diversity, we further investigated the molecular characteristics by comparing sequence similarities of nine full-length PDCoV genomes from China as representative strains of each of the subgenogroups with PDCoV AH2019-H (GenBank ID: MN520198.1) in GI-a as a query strain (Fig. 3), using SimPlot ver.3.5.1 software. The PDCoV Spike ORF revealed the lowest similarity levels (< 93%), while the other genome sequences demonstrated a greater similarity (> 95%). It is important to emphasize that NS6 gene of CHzmd2019 (GenBank ID: MN781985.1) exhibited a low similarity level (< 87%). Consistently with the results of the phylogenetic trees, the spike ORF of PDCoVs from different subgenogroups is highly variable, while the ORF encoding other nonstructural (pp1a/b) is relatively conserved (Fig. 3), clarifying the obtained tree results using the spike ORF genomic fragment (Fig. 2).

### The landscape of amino acid variations of structural proteins during 2004–2020

**Fig. 4:**
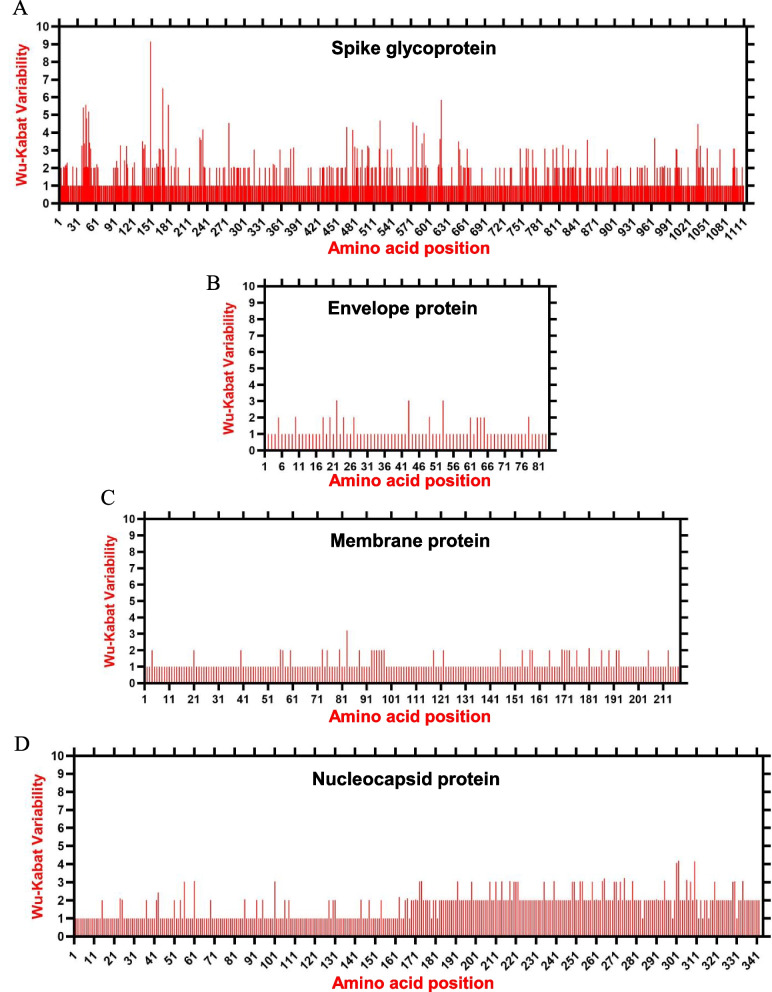
The landscape of amino acid variation during 2004–2020 determined by Wu-Kabat amino acids variability plotting. **A** Spike glycoprotein, **B** Envelop protein, **C** Membrane protein, **D** Nucleocapsid protein. The earliest isolated virus, CHN-AH-2004 (GenBank ID: KP757890.1), was used as a query. Y-axes represent the Wu-Kabat variability coefficient values, where the estimation limit is “1”. Above the limit “> 1” represents variations. X-axes represent the amino acid positions relative to the corresponding protein

The structural proteins of PDCoV, including S, E, M, and N, were further assessed for amino acid variations during 2004–2020 using the Wu-Kabat coefficient implemented by the PVS (Protein Variability Server) due to their potential role in vaccine development. The earliest reported strain, CHN-AH-2004 (GenBank ID: KP757890.1), isolated in China in 2004, was selected as a query to make the consensus amino acids sequence of all 166 strains. The consensus sequence for S, E, M, and N consisted of 1110 aa, 83 aa, 217 aa, and 342 aa, respectively, according to the Wu-Kabat variability method (Fig. 4A, B, C, and D respectively). The S glycoprotein was the most highly variable, especially for the N-terminal region (Fig. 4A). In contrast, the structure proteins E, M, and N are observed to be relatively conserved (Fig. 4B, C, and D, respectively), which is consistent with the results of genomic similarity analysis of representative viruses (Fig. 3).

### Recombination pattern of PDCoV full-length genomes

To further elucidate the genetic variability patterns of PDCoVs and thoroughly understand the risk of a zoonotic emergence, we explored the occurrence of recombination between the complete genome strains. The genomic recombination in coronaviruses is a common event required for normal replication and rapid spread [69]. Herein, we analyzed a total of 166 PDCoV full-length genomes to assess the recombination patterns, the parental sequences and the mapping of possible breakpoints using the seven distinct algorithms of the RDP4 software package [65]. We identified 31 potential recombination events (Table 2, Supplementary Fig. 5), among which 19 were intragenogroup, including 15 between strains from G.I. clade (Events 12–13, Events 17–24, Events 26–28, Events 30–31) and four between strains from GII clade (Events 5,14, 25 and 29), 12 other recombinants were intergenogroup and occurred between GI and GII clades (Events 1–4, Events 6–11, Events 15–16). Interestingly, most recombinants were identified in China or involved at least one China PDCoV parental sequence (Table 2). Among the 31 recombination events, 19 recombinants resulted from recombination between China strains and seven between China and Southeast Asian strains (Table 2). Importantly, the PDCoV/Haiti/Human/ 0256–1/2015 (GenBank ID: MW685623.1) that has been reported infecting humans in Haiti is identified in our analysis as a recombinant (Event 20), resulting from recombination between China strain AH2019-H (GenBank ID: MN520198.1) and the United States strain USA/Arkansas61/2015 (GenBank ID: KR150443.1) as parental major and minor sequences, respectively (Table 2). China strain AH2019-H (GenBank ID: MN520198.1) is also involved in the recombination event 28, CHN-GD16–05 (GenBank ID: KY363868.1) as a major parent (Table 2). Therefore, the recombination frequency of China strains is extremely high and extensive, posing a highly probable risk of human infection. As shown in Supplementary Fig. 5, the beginning or ending breakpoints of 12 recombination events are located within or closer to the spike ORF region; however, the recombination can occur all over the PDCoVgenome. For example, the genomic regions encoding PL-pro (in event 15), 3CLpro (in events 11, 21, 22, 23, and 29), RNA-dependent RNA polymerase (RdRp, in events 14 and 21), and nsp14 (in events 5, 8, 12, 17, 18, 24, and 27) were all involved in recombination.

**Table 2:** Identification of 31 potential recombination events in the complete genome of PDCoVs isolated during 2004–2020. The genogroups were defined based on the phylogenetic tree of PDCoV full-length genome ine in this study (see Fig. 1). The *P* values for each detection method are shown in Supplementary Table 1

Event serial number	Recombinant		Minor parent		Major parent	
	GenBank ID: Virus name/Country-Year	Genogroup	GenBank ID: Virus name/Country-Year	Genogroup	GenBank ID: Virus name/Country-Year	Genogroup
1	KY363867.1:CHN-GD16–03(China-2016)	GI-d	MN025260.1:CH/GX/1468B/2017 (China-2017)	GII-a	MF280390.1:CHN-GD-2016(China-2016)	GI-d
2	MN781985.1:CHzmd2019(China-2019)	GI-a	KX361344.1:P2_13_ST2_0313/PDCoV/0213/Thailand (Thailand-2013)	GII-a	MZ802955.1:CH-HLJ-20(China-2020)	GI-a
3	KX998969.1:P29_15_VN_1215(Viet_Nam-2015)	GII-a	KR265859.1:USA/Minnesota159/2014(USA-2014)	GI-a	KX834351.1:PDCoV/Swine/Vietnam/HaNoi6/2015(Viet_Nam-2015)	GII-a
4	KT266822.1:CH/Sichuan/S27/2012(China-2012)	GI-g	KX361344.1:P2_13_ST2_0313/PDCoV/0213/Thailand(Thailand-2013)	GII-a	KP757891.1:CHN-HB-2014(China-2014)	GI-b
5	*MN173781.1:CHN-GX81–2018(China-2018)	GII-b	KX834351.1:PDCoV/Swine/Vietnam/HaNoi6/2015(Viet_Nam-2015)	GII-a	MN173779.1:CHN-GX11–2018(China-2018)	GII-b
6	*MK572803.1:SCNC201705(China-2017)	GI-f	KX998969.1:P29_15_VN_1215(Viet_Nam-2015)	GII-a	MK211169.1:CHN/Sichuan/2017(China-2017)	GI-f
7	*MF642325.1:CHN/QH/2017/1(China-2017)	GI-b	KX834351.1:PDCoV/Swine/Vietnam/HaNoi6/2015(Viet_Nam-2015)	GII-a	MN520196.1:SD2018–306(China-2018)	GI-g
8	KY065120.1:CHN/Tianjin/2016(China-2016)	GI-d	MN025260.1:CH/GX/1468B/2017 (China-2017)	GII-a	MH715491.1:PDCoV/CHGD/2016(China-2016)	GI-d
9	MN520209.1:JS2019-A1414(China-2019)	GI-d	KX998969.1:P29_15_VN_1215(Viet_Nam-2015)	GII-a	MW854634.1:104–553(Taiwan-2015)	GI-d
10	MN520209.1:JS2019-A1414(China-2019)	GI-d	MN025260.1:CH/GX/1468B/2017 (China-2017)	GII-a	KT021234.1:CH/SXD1/2015(China-2015)	GI-d
11	KT021234.1:CH/SXD1/2015(China-2015)	GI-d	MZ802774.1:PDCoV/CBR-3/2016/Thailand(Thailand-2016)	GII-a	KU665558.1:CHN-LYG-2014(China-2014)	GI-d
12	*MN520196.1:SD2018–306(China-2018)	GI-g	MT260149.1:HNZK-04-P5(China-2018)	GI-d	MG242062.1:CHN-HeB1–2017(China-2017)	GI-e
13	*KT021234.1:CH/SXD1/2015(China-2015)	GI-d	MN781985.1:CHzmd2019(China-2019)	GI-a	MH715491.1:PDCoV/CHGD/2016(China-2016)	GI-d
14	*MK993519.1:CHN/Sichuan/2019(China-2019)	GI-f	MN025260.1:CH/GX/1468B/2017 (China-2017)	GII-a	MN173781.1:CHN-GX81–2018(China-2018)	GII-b
15	MF095123.1:CHN-HG-2017(China-2017)	GI-d	KX998969.1:P29_15_VN_1215(Viet_Nam-2015)	GII-a	KR265849.1:USA/Michigan447/2014(USA-2014)	GI-a
16	KX443143.2:CH-01(China-2016)	GI-g	KX361344.1:P2_13_ST2_0313/PDCoV/0213/Thailand(Thailand-2013)	GII-a	LC260043.1:OKN/JPN/2014(Japan-2014)	GI-a
17	*MF280390.1:CHN-GD-2016(China-2016)	GI-d	KX443143.2:CH-01(China-2016)	GI-g	MH715491.1:PDCoV/CHGD/2016(China-2016)	GI-d
18	MK355396.1:CHN-SC2015(China-2016)	GI-f	MT260149.1:HNZK-04-P5(China-2018)	GI-d	MK211169.1:CHN/Sichuan/2017(China-2017)	GI-f
19	MK211169.1:CHN/Sichuan/2017(China-2017)	GI-f	MN520206.1:HN2019-C132(China-2019)	GI-d	MN520207.1:JS2018-QF49(China-2018)	GI-f
20	*MW685623.1:PDCoV/Haiti/Human/0256–1/2015(Haiti-2015)	GI-a	KR150443.1:USA/Arkansas61/2015(USA-2015)	GI-a	MN520198.1:AH2019-H(China-2019)	GI-a
21	*MN520207.1:JS2018-QF49(China-2018)	GI-f	MK005882.1:Swine/CHN/SC/2018/1(China-2018)	GI-f	KU665558.1:CHN-LYG-2014(China-2014)	GI-d
22	MT663769.1:CHN-TS1–2019(China-2019)	GI-c	MK211169.1:CHN/Sichuan/2017(China-2017)	GI-f	MF041982.1:SHJS/SL/2016(China-2016)	GI-e
23	*MN520206.1:HN2019-C132(China-2019)	GI-d	MK211169.1:CHN/Sichuan/2017(China-2017)	GI-f	KU665558.1:CHN-LYG-2014(China-2014)	GI-d
24	*MF041982.1:SHJS/SL/2016(China-2016)	GI-e	KP757892.1:CHN-JS-2014(China-2014)	GI-d	MG242062.1:CHN-HeB1–2017(China-2017)	GI-e
25	MN173781.1:CHN-GX81–2018(China-2018)	GII-b	MN025260.1:CH/GX/1468B/2017 (China-2017)	GII-a	MN520199.1:AH2018–93(China-2018)	GII-b
26	MN520206.1:HN2019-C132(China-2019)	GI-d	MK572803.1:SCNC201705(China-2017)	GI-f	MN520190.1:AH2018–322(China-2018)	GI-d
27	MN520204.1:HN2018-LH2(China-2018)	GI-f	MN520190.1:AH2018–322(China-2018)	GI-d	MK572803.1:SCNC201705(China-2017)	GI-f
28	*KY363868.1:CHN-GD16–05(China-2016)	GI-a	KX443143.2:CH-01(China-2016)	GI-g	MN520198.1:AH2019-H(China-2019)	GI-a
29	*MK993519.1:CHN/Sichuan/2019(China-2019)	GI-f	MN025260.1:CH/GX/1468B/2017 (China-2017)	GII-a	MN520199.1:AH2018–93(China-2018)	GII-b
30	*MT263013.1:CHN-HN-17(China-2017)	GI-g	MK572803.1:SCNC201705(China-2017)	GI-f	MK005882.1:Swine/CHN/SC/2018/1(China-2018)	GI-f
31	MN520194.1:SD2018–10(China-2018)	GI-c	LC260043.1:OKN/JPN/2014(Japan-2014)	GI-a	MH025764.1:CH/JXJGS01/P50(China-2016)	GI-c

* The major or minor parent may be the actual recombinant due to the possibility of misidentification

To further confirm the recombination authenticity, we performed an ML phylogenetic analysis of PDCoV strains implicated in the recombination events based on different genomic fragments. The phylogenetic trees exhibited that the recombinant strains are more closely related to the major or minor parents in the designated regions, concordant with the recombination analysis findings. For example, in Event 1, the nt 1–3000 region of the recombinant CHN-GD16–03 is genetically closer to the major parent CHN-GD-2016 (Supplementary Fig. 6A), whereas the nt 19,000–25,000 region of CHN-GD16–03 is genetically closer to the minor parent CH/GX/1468B/2017 (Supplementary Fig. 6B), indicating that our detected recombination events are real.

### Antigenic drift of PDCoV spike glycoprotein

**Fig. 5:**
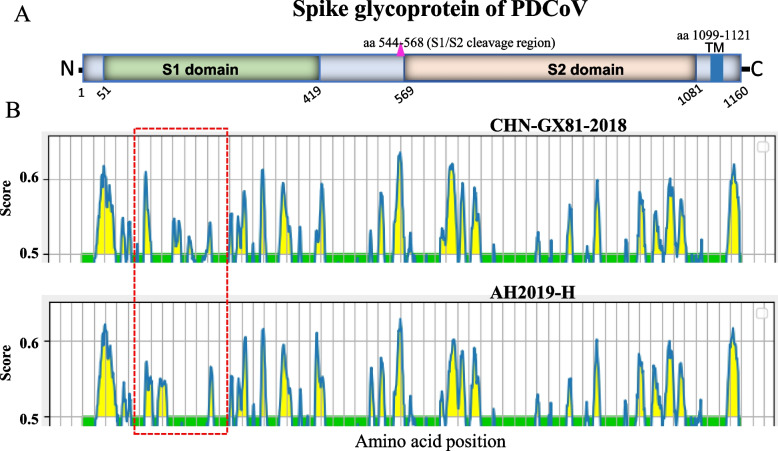
Linear B cell epitope distribution map of the predicted PDCoV S glycoprotein. **A** Domain structure of PDCoV S glycoprotein, including S1 domain (aa51–419), S2 domain (aa569–1081), and transmembrane domain (TM, aa1099–1121). **B** Predicted linear B cell epitopes in the S glycoprotein of representative strain AH2019-H in the GI and CHN-GX81–2018 in the GII using the BepiPred-2.0 server. The horizontal axis represents the corresponding position of the S glycoprotein amino acid sequence, and the vertical axis represents the residue scores. The potential epitopes are residue scores above the threshold of 0.5 and are represented by yellow color. Regions with large epitope differences between the two representative strains are boxed in red

**Fig. 6:**
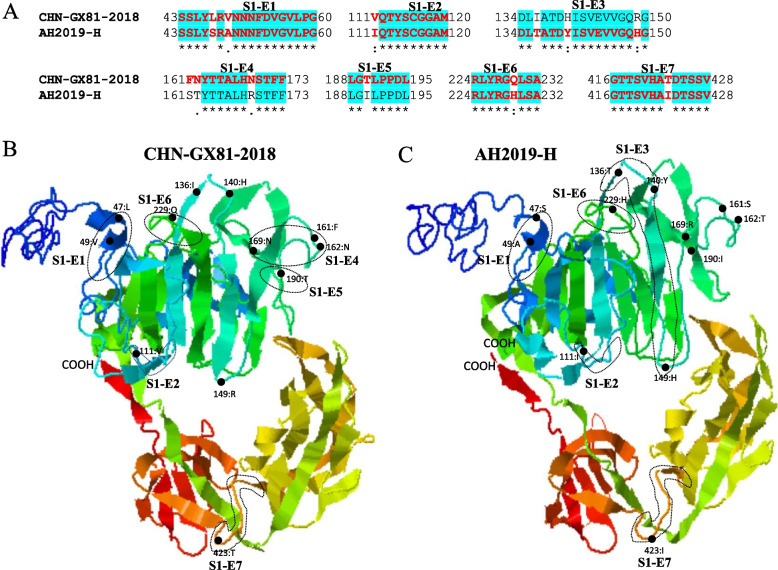
Comparison of linear B cell epitope structures and corresponding amino acids of the S1 domain of AH2019-H and CHN-GX81–2018 representative strains. **A** Amino acid sequences corresponding to linear B cell epitopes of the S1 domain. The amino acid sequences of predicted linear B-cell epitopes are shown in red letters. Blue highlights indicate identical amino acid sequences in predicted epitopes between the two representative strains. The numbers on both sides of the amino acid sequences indicate the beginning and ending amino acid positions in the peptide segment relative to the S glycoprotein of corresponding viruses. **B** Three-dimensional structural map of epitopes in the S1 domain of the S glycoprotein of virus CHN-GX81–2018. The position of the epitope in the three-dimensional structural map is circled by a dashed circle. Black dots indicate sites of amino acid variation. **C** Three-dimensional structural map of epitopes in the S1 domain of the S glycoprotein of AH2019-H virus

Given the hypervariability of the viral spike glycoprotein encoding genomic region and the crucial role of spike glycoprotein in binding receptors and inducing the generation of neutralizing antibodies, we find it necessary to analyze the antigenic differences of S glycoprotein between the two PDCoV genogroups GI and GII involving AH2019-H (GenBank ID: MN173781) and CHN-GX81–2018 (GenBank ID: MN520198.1) as representative strains since they displayed the farthest genetic distance in the phylogenetic tree (Fig. 1, Supplementary Fig. 1). As shown in Fig. 5, we identified a significant difference in the distributions of the predicted linear B cell epitope in the RBD of the S1 domain (aa 51–419). Subsequently, we mapped the amino acid variations to the B cell epitopes on the three-dimensional structures of the S1 domain of the two PDCoV representative S glycoproteins using the Iterative Thread ASSEmbly Refinement server (I-TASSER) [68]. The 3D PDCoV S1 structural model revealed that three of seven predicted epitopes in the S1 domain underwent significant amino acid substitutions, of which three amino acid mutations (136 T, 140Y, and 149H) resulted in a new epitope (S1-E3) of AH2019-H, three amino acids (161ST162 and 169R) and one amino acid mutation (190I) resulted in the loss of the epitopes (S1-E4) and (S1-E5) of AH2019-H, respectively. The structures of the remaining four predicted epitopes might not be significantly affected by amino acid variation (Fig. 6). The B cell epitopes’ findings require more experimental investigation.

## Discussion

Presently, there is no adopted PDCoV genotyping taxa classification, and few are researchers who tried to phylogenetically classify the virus. Sun et al., using the neighbor-joining (NJ) method analysis of 29 PDCoV spike ORF nucleotide sequences, divided the virus into three lineages, including the China lineage, the USA/Japan/Korea lineage, and the Vietnam/Laos/Thailand lineage [53]. Meanwhile, He et al., based on 119 PDCoV full-length genomes, sorted PDCoVs into four lineages: Thailand, early China, America, and China lineages, using the SplitsTree5 software with the Kimura 2-parameter model [8]. Herein, we attempted to establish a more reliable PDCoV phylogenetic classification using the maximum likelihood approach (ML) in the IQ-TREE [61] with the best fitting models for PDCoV based on an extensive number of full-length genome sequences (*n* = 166 isolates), complete ORF1a/b nucleotide sequences (*n* = 166), complete ORF spike sequences(*n* = 166), and complete ORFs E-N sequences (*n* = 166). The generated tree based on PDCoV full-length genomes revealed two main PDCoV genogroups (GI and GII) with distinct geographic characters. GI and GII are further subdivided into seven and two subgenogroups (a-g, a-b), respectively, where China strains are distributed in all subgenogroups. Congruently, the phylogenetic tree based on the complete individual gene ORF1a/b revealed that PDCoV strains cluster into two main genogroups (GI and GII) with seven and two further subgenogroups, respectively (Fig. 2A, Supplementary Fig. 2), supporting the proposed genotyping classification (Fig. 1). Our phylogenetic tree evinces that two Human PDCoVs (0081–4 and 0329–4) cluster together into GI-d genetically close to CHN/Tianjin/2016, and Human PDCoV (0256–1) clusters into GI-a subclade close to PDCoV/USA/Arkansas61/2015(GenBank ID: KR150443.1) (Supplementary Fig. 1). Characterizing the PDCoV subgenogroups will facilitate the research communication and the prediction of future new lineages. Therefore, our proposed genotyping is insightful for mapping the geographical distribution and predicting the risk of human infection and genetic changes.

We assessed the amino acid variations by comparing the earliest reported virus, CHN-AH-2004 (GenBank ID: KP757890.1), isolated in China in 2004, with the remaining 165 PDCoV strains. The analysis revealed extensive variations in the S glycoprotein. The N protein was also observed to have significant variations across their amino acids, while the envelope and membrane proteins showed relatively lower variations (Fig. 4A, B, C, and D).

Recombination is a frequent event affecting coronavirus genomes and driving their intraspecies diversity with great potential to engage host receptors for interspecies transmission, hence genome adaptation and emergence of novel CoV variants [70]. The precise mechanisms behind the recombination among CoVs are not properly understood; however, its occurrence is highly suggested during the replication phase and is driven by homologous RNA recombination mediated by a copy choice mechanism [71]. Genetic recombination has been reported for both animal and human CoVs, including the porcine epidemic diarrhea virus (PEDV) [72], OC43 [73], and SARS-CoV [74]. The PDCoV has been demonstrated in multiple studies as having a large and complex diversity with the potential to cross interspecies barriers in a variety of avian [69] and mammalian [7] species.

The first identified PDCoV HKU15 was demonstrated, resulting from a recombination between sparrow deltacoronavirus HKU17 (SpCoV HKU17)/bulbul coronavirus HKU11 (BuCoV HKU11) [69], and DCoV QuaCoV UAE-HKU30 from recombination between porcine coronavirus HKU15 (PorCoV HKU15)/sparrow coronavirus HKU17 (SpCoV HKU17) and munia coronavirus HKU13 (MunCoV HKU13) [69]. The intra-lineage recombination analysis of China and early China PDCoV lineages by He et al. has located the recombination breakpoints within the ORF1a/b region and indicated that recombination is lacking in the USA strains [8]. Recently, Zhao et al. have reported by analyzing the S glycoprotein of CHN-SC2015(GenBank ID: MK355396.1) and its complete genome multiple insertion-deletion variations across CHN-SC2015 genomes, including a 3-nt deletion in the S gene, a 6-nt and 9-nt insertion in the ORF1ab gene compared to strains from the USA and Asia, and 11-nt deletion in 3’UTR [75]. Zhao et al. demonstrated that CHN-SC2015 resulted from recombination between TT_1115 (GenBank ID: KU984334) and SHJS/SL/2016 (GenBank ID: MF041982) as minor and major parental sequences, respectively, with breakpoints mapped at nt 6020 and nt 7069 corresponding to the nsp3 and nsp4 coding regions [75]. Furthermore, Lednicky et al. have reported that Haitian human PDCoV (Hu-PDCoV) strains 0081–4 and 0329–4 show a great similarity (~ 99.97%) and are closely related to China strain CHN/Tianjin/2016 (99.8%), while the variant 0256-1was suggested related to a strain detected in Arkansas, USA, in 2015 [27]. In our analysis, CHN-SC2015 is identified as resulting from recombination between HNZK-04-P5 (GenBank ID: MT260149.1) and CHN/Sichuan/2017 (GenBank ID: MK211169.1) as minor and major parental sequences, respectively, with breakpoints located at nt 15,765 and nt 18,968, within ORFa/b. In addition, we found that Spike genomic region is a PDCoV recombination hotspot and identified that strains from the USA are involved in the recombination, more importantly, in a recombinant isolated from human PDCoV/Haiti/Human/0256–1/2015 (GenBank ID: MW685623.1, Event 20) that exhibited breakpoints around the Spike gene, which is in consistence with the report of Nikolaidis et al. displaying the occurrence of genomic exchanges by nonhomologous recombination between CoVs in the neighborhood of the Spike protein [76]. Interestingly, Nikolaidis et al. demonstrated substantial recombination between CoVs of different subgenera, revealing the instability of the Spike ORF coding region. Nikolaidis et al. reported that CoVs gain or exchange their accessory ORFs by nonhomologous recombination with different CoV genera and different viruses involving regions surrounding the spike protein [76]. Additionally, another study reported that canine coronaviruses CCoVs II, with potential double recombinant origins involving partial S gene exchange with TGEV, have been identified in dogs with gastroenteritis [77]. The analysis of 3′-end genomic region of the four identified recombinant viruses and the nearly complete genome of two of the four strains indicated the existence of TGEV-like CCoVs recombinants. Therefore, TGEVs, that arose from CCoVs gave rise to TGEV-like CCoVs through recombination [77]. Given that human coronaviruses exhibited genetic exchanges with coronaviruses of zoonotic origin and considering the high rate of recombination among CoVs, particularly wild animal populations that are in permanent contact, an effective preparedness for the next unpredictable human CoV outbreak is highly necessary [78]. Herein, in the recombinant event 20, human PDCoV/Haiti/Human/0256–1/2015 seems to involve a combination of the Spike protein of the “USA/Arkansas61/2015” strain (Genbank ID: KR150443.1) with that of ORFa/b of a strain from China. Structurally, the Spike proteins of Alpha(α)- and Delta(δ)- CoVs have been reported to be highly similar [39], and more recently, the Spike-based-phylogenetic tree in Nikolaidis et al. report revealed that all δ- CoVs and most of the α-CoVs established a single group [76], suggesting that the common ancestor of δ- CoVs has gained, through recombination, its Spike protein from the α-CoVs. Therefore, the extensive recombination among strains from China, their involvement in the recombination event of the human Haiti strain, and their high genetic relatedness with Human PDCoV speculate an increased risk for viral cross-species transmission and adaptation to new hosts, including humans.

A recent report emphasized the importance of NS6 in PDCoV vaccine design, suggesting NS6-deficient mutant viruses as promising live-attenuated vaccine candidates [36]. Zhang et al. recommended the NSP6 protein as an important PDCoV virulence and pathogenicity factor when deletion of the NS6 gene resulted in an attenuated viral phenotype [36]. Accordingly, we demonstrated that NS6 of strain CHzmd2019 (GenBank ID: MN781985.1) displayed the highest dissimilarity (Fig. 2), which is supported by further recombination mapping evidence showing the breakpoints within the NS6 coding region at nt 23,506 and nt 23,987 (Event 2, Supplementary Fig. 5). Here, we highlight the need for more research to investigate the role of PDCoV NS6 accessory protein, which remains unclear.

Spike glycoprotein mutations play a crucial role in the pathogenesis and host tropism of PDCoV and the other coronavirus genus [79]. Liu et al. divided the S1 subunit of PDCoV into four domains: S1(A-D), which strongly induce the neutralizing antibodies, especially S1A and S1B [80]. In addition, He et al. confirmed that five positive selections of amino acids in the spike glycoprotein contribute to a more efficient entry of PDCoV into host cells [8]. Herein, we exhibit significant differences in the spike region between PDCoV strains (Figs. 3 and 4A) and suggest a significant antigenic drift in the RBD of S1 domain during the prediction of linear B-cell epitopes of S glycoprotein of the two representative strains CHN-GX81–2018 and AH2019-H (Figs. 5 and 6). Therefore, the significant amino acid variation might be involved in the evolutionary diversity of PDCoVs and in evading the immune system of host cells.

In summary, our report proposes a more reliable PDCoV genotyping classification system and shows that extensive recombination and genomic mutations in PDCoV strains from China are behind the genetic variability and the global distribution of this virus. The genetic relatedness and implication of strains from China in the generation of human Haiti recombinant strain ring bells on the zoonotic risk and future PDCoVs cross-species transmission and human infection. The proposed PDCoV genotyping classification might help predict new PDCoV lineages and would be insightful in preventing the risk of animal-human spillover and promoting PDCoV prevention control strategies.

## Supplementary Information

**Additional file 1.**

**Additional file 2.**

**Additional file 3.**

**Additional file 4.**

**Additional file 5.**

**Additional file 6.**

**Additional file 7.**

## Data Availability

The nucleotide and protein sequence data used in this study are mainly available on GenBank (https://www.ncbi.nlm.nih.gov/nuccore/?term=porcine+deltacoronavirus%2C+complete+genome).
