# Supplementary material for: Evolutionary plasticity of zoonotic porcine Deltacoronavirus (PDCoV): genetic characteristics and geographic distribution
Source: BMC Vet Res. 2022 Dec 22;18:444. doi: 10.1186/s12917-022-03554-4 (PMC9772601; doi:10.1186/s12917-022-03554-4)

Evolutionary Plasticity of Zoonotic Porcine Deltacoronavirus (PDCoV): Genetic Characteristics and Geographic Distribution

Amina Nawal Bahoussi[1#], Pei-Hua Wang[1#], Pir Tariq Shah[1#], Hongli Bu[2], Changxin Wu[1,3,4,5] *, Li Xing[1,3,4,5]*

Supplementary Figure 2. The phylogenetic tree based on ORF 1a/b complete nucleotide sequences of 166 PDCoVs. Multiple sequence alignment was performed using ClustalW. The Maximum Likelihood phylogenetic trees (ML) were constructed in IQ-TREE multicore version 1.6.12 using the best-fitting model GTR+F+I+G4 with 1000 bootstraps. The viruses identified in China are indicated with red branches. The viruses reported in China but falling into full-length genome-based GI-a and GII-a subgroups are shown in red branches with red circles. The human Haiti PDCoVs are indicated with blue circles. The scale bar in the bottom left represents nucleotide substitutions per site. Strains are formatted as GenBank accession number_virus name_country-year of collection_.

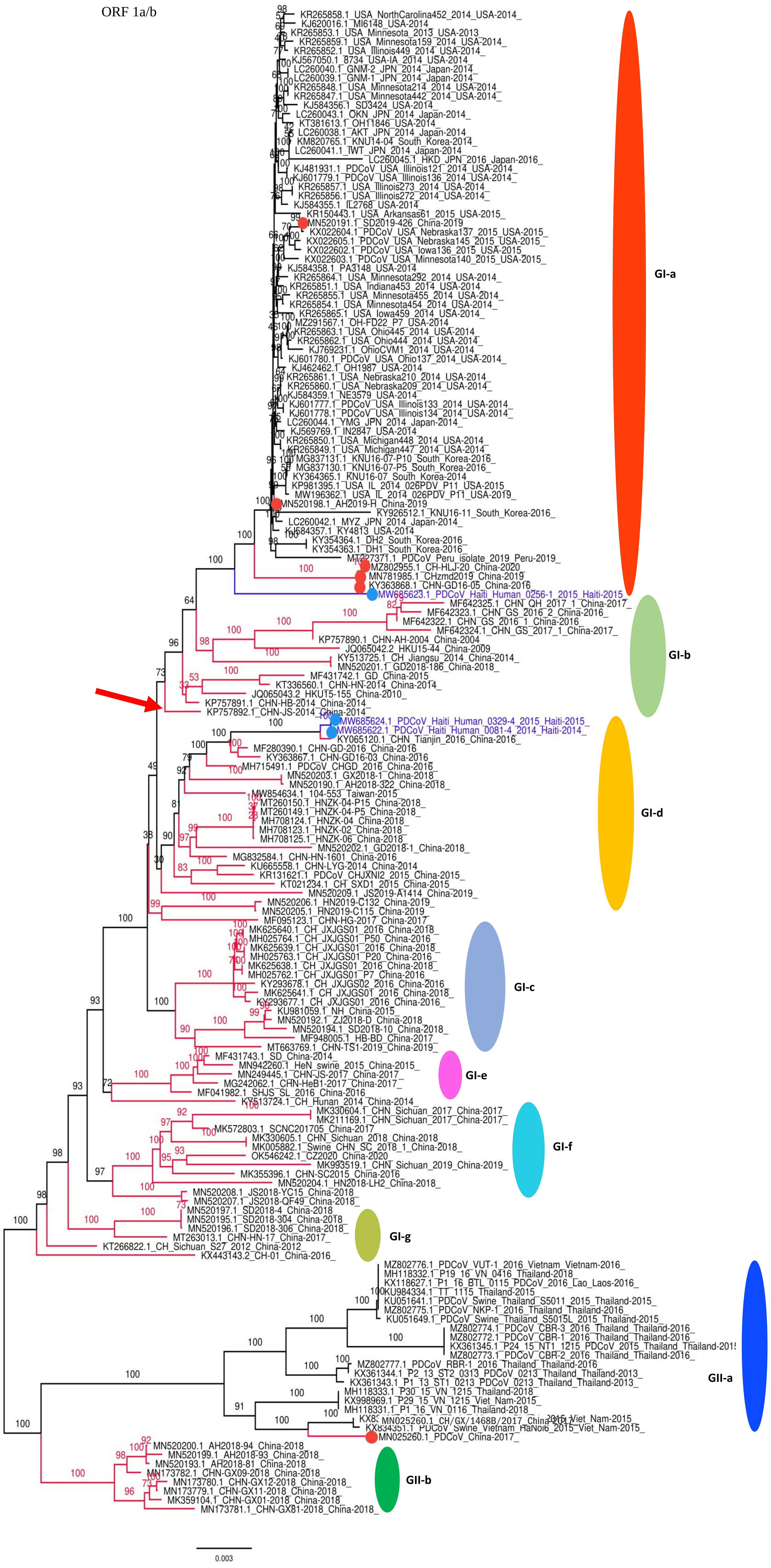

Supplement: Supplementary file 2 — Additional file 2. [file 12917_2022_3554_MOESM2_ESM.pdf]
