# Supplementary material for: Evolutionary plasticity of zoonotic porcine Deltacoronavirus (PDCoV): genetic characteristics and geographic distribution
Source: BMC Vet Res. 2022 Dec 22;18:444. doi: 10.1186/s12917-022-03554-4 (PMC9772601; doi:10.1186/s12917-022-03554-4)

Evolutionary Plasticity of Zoonotic Porcine Deltacoronavirus (PDCoV): Genetic Characteristics and Geographic Distribution

Amina Nawal Bahoussi[1#], Pei-Hua Wang[1#], Pir Tariq Shah[1#], Hongli Bu[2], Changxin Wu[1,3,4,5] *, Li Xing[1,3,4,5]*

Supplementary Figure 5. Recombination analysis of 166 PDCoVs. **(A)** The open reading frames (ORFs) contained in the full-length genome sequence of PDCoVs are ORF1a/b, Spike ORF, envelope protein ORF (E), membrane ORF (M), NS6 ORF (NS6), nucleocapsid protein ORF (N), and NS7 ORF (NS7) from the 5' terminus to the 3' terminus of the genome. The genomic regions in ORF 1a/b encoding papain-like protease (PL-pro in nsp3), 3C-like protease (3CLpro, nsp5), RNA-dependent RNA polymerase (RdRp, nsp12), and nsp14 were indicated. **(B)** 31 potential recombination events were identified using the recombination detection program (RDP4) based on the nucleotide sequences of the full-length genomes of 166 PDCoVs. The left side shows the serial number of the recombination events and the GenBank accession ID, strain name, collection country and year of the recombinant. The green and pink blocks on the right represent recombination regions of the major and minor parents, respectively, with strain names. Numbers indicate nucleotide positions relative to the beginning and ending breakpoints of the recombination.

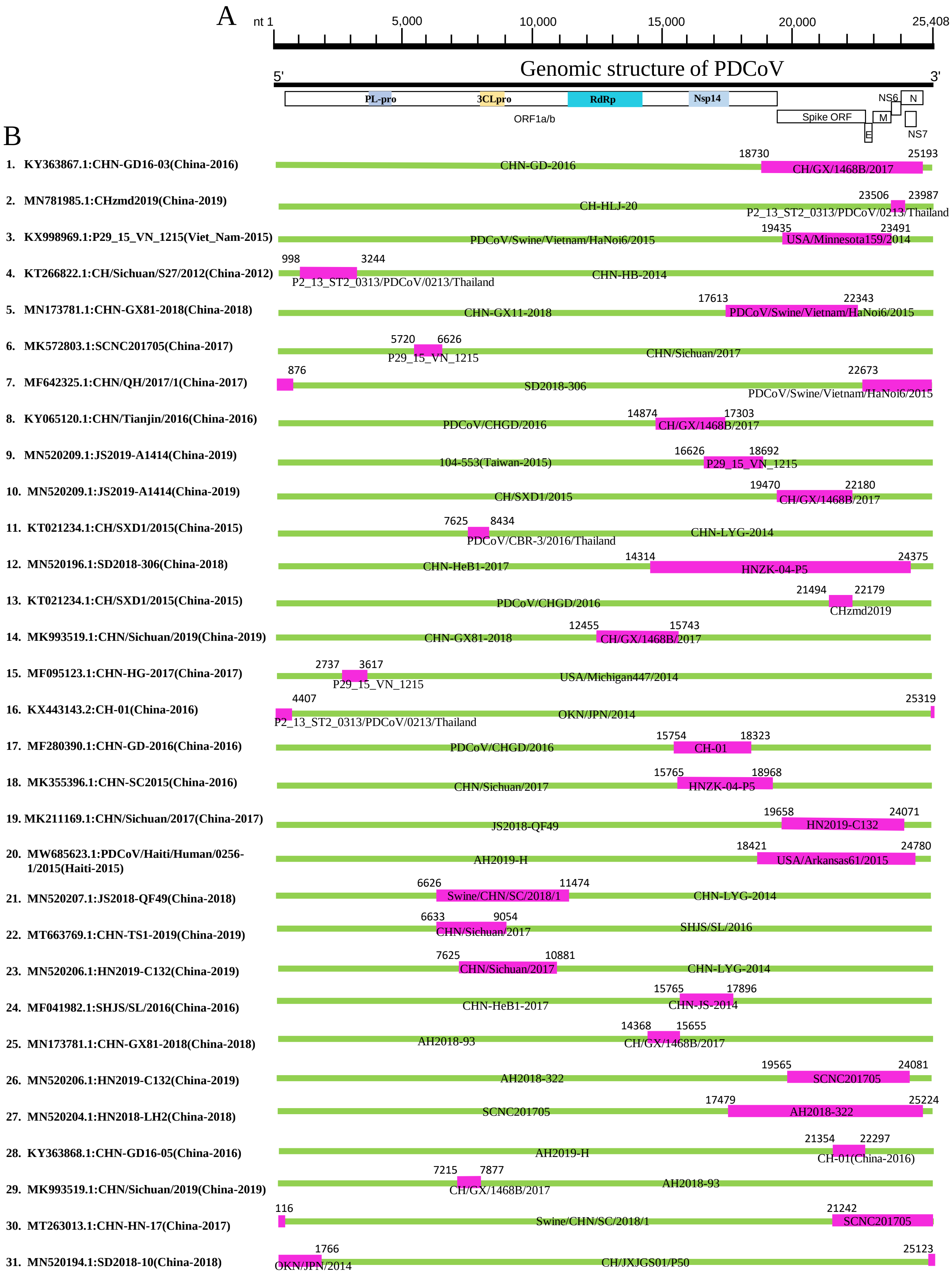

Supplement: Supplementary file 5 — Additional file 5. [file 12917_2022_3554_MOESM5_ESM.pdf]
