# Supplementary material for: Evolutionary plasticity of zoonotic porcine Deltacoronavirus (PDCoV): genetic characteristics and geographic distribution
Source: BMC Vet Res. 2022 Dec 22;18:444. doi: 10.1186/s12917-022-03554-4 (PMC9772601; doi:10.1186/s12917-022-03554-4)

Evolutionary Plasticity of Zoonotic Porcine Deltacoronavirus (PDCoV): Genetic Characteristics and Geographic Distribution

Amina Nawal Bahoussi[1#], Pei-Hua Wang[1#], Pir Tariq Shah[1#], Hongli Bu[2], Changxin Wu[1,3,4,5] *, Li Xing[1,3,4,5]*

Supplementary Figure 6. Phylogenetic trees based on the nt 1–3,000 **(A)** and nt 19,000–25,000 **(B)** regions of the full-length genomes of 48 PDCoVs comprising strains involved in recombination events 1, 3, 4, 12, 19 and 27 using the Maximum Likelihood method in MEGA-11 software. Different graphs represent isolates involved in different recombination events. Red represents the recombinant strain, yellow represents the major parent, and blue represents the minor parent. The numbers on each branch represent bootstrap values for 1000 iterations. The scale bar in the bottom left represents 0.005 nucleotide substitutions per site. Strains are formatted as GenBank accession number: virus name (country-year of collection).

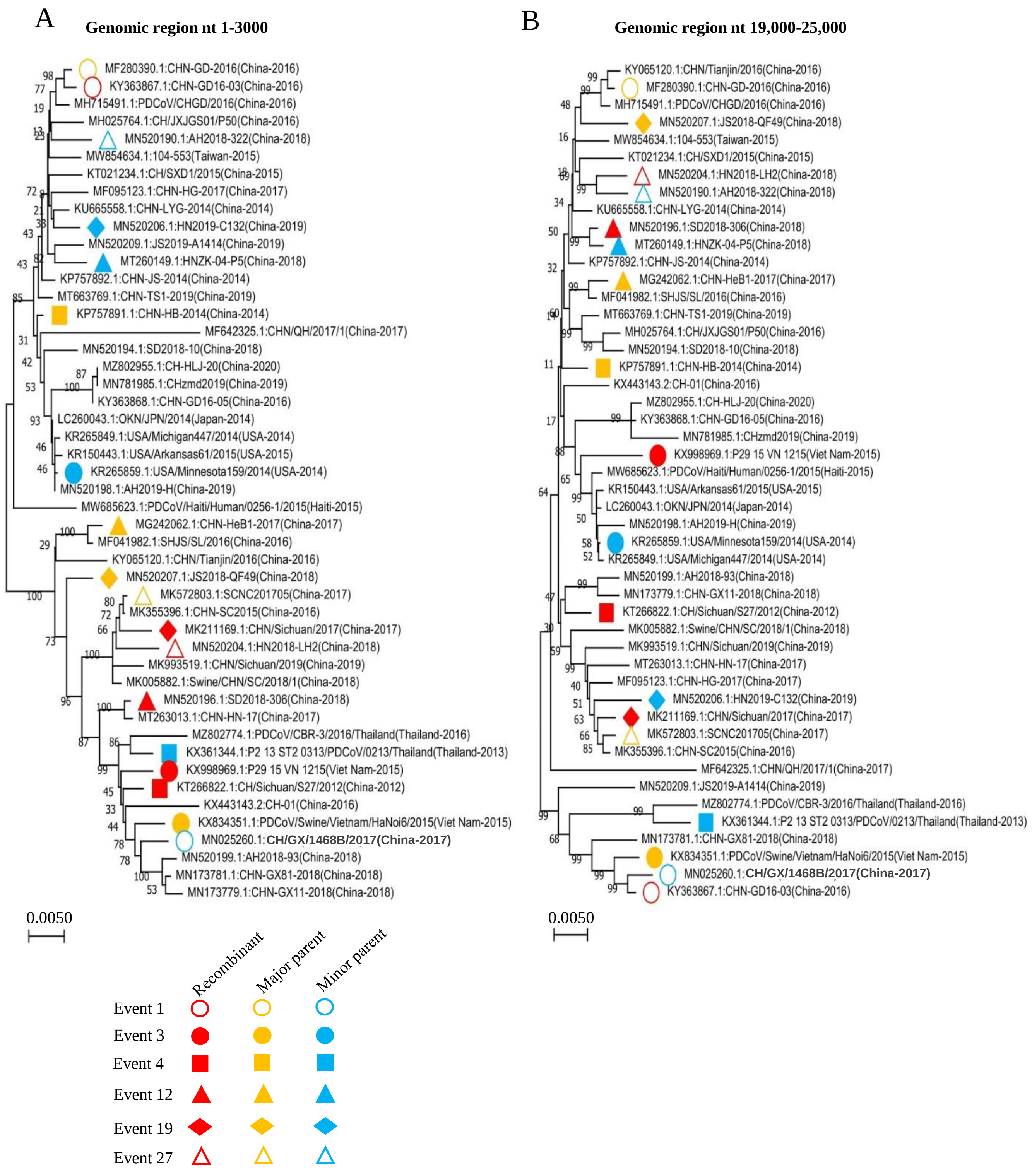

Supplement: Supplementary file 6 — Additional file 6. [file 12917_2022_3554_MOESM6_ESM.pdf]
