# Supplementary material for: Evolutionary plasticity of zoonotic porcine Deltacoronavirus (PDCoV): genetic characteristics and geographic distribution
Source: BMC Vet Res. 2022 Dec 22;18:444. doi: 10.1186/s12917-022-03554-4 (PMC9772601; doi:10.1186/s12917-022-03554-4)
Supplement: Supplementary file 7 — Additional file 7. [file 12917_2022_3554_MOESM7_ESM.pdf]

Evolutionary Plasticity of Zoonotic Porcine Deltacoronavirus (PDCoV): Genetic Characteristics and Geographic Distribution

Amina Nawal Bahoussi[1#], Pei-Hua Wang[1#], Pir Tariq Shah[1#], Hongli Bu[2], Changxin Wu[1,3,4,5*], Li Xing[1,3,4,5]*

Supplementary Table 1. *P* value of each recombination detection method*

Recombination Event serial number	P value						
	RDP	ENECONV	Bootscan	Maxchi	Chimaera	SiSscan	3Seq
1	$2.31x10^{-30}$	$2.63x10^{-48}$	NS	$3.18x10^{-23}$	$4.97x10^{-23}$	$3.75x10^{-23}$	$2.46x10^{-11}$
2	$7.28x10^{-34}$	$2.64x10^{-26}$	NS	$1.00x10^{-06}$	$9.85x10^{-07}$	NS	$4.93x10^{-11}$
3	$7.70x10^{-32}$	$6.87x10^{-28}$	$5.96x10^{-20}$	$1.40x10^{-14}$	9.18x10-^{16}	$5.79x10^{-16}$	$2.46x10^{-11}$
4	$1.48x10^{-29}$	$9.67x10^{-24}$	$1.71x10^{-17}$	$9.33x10^{-12}$	$6.83x10^{-13}$	$2.44x10^{-10}$	$2.46x10^{-11}$
5	$7.32x10^{-21}$	$3.72x10^{-09}$	$4.65x10^{-07}$	$1.22x10^{-14}$	$5.07x10^{-16}$	$3.86x10^{-09}$	$2.46x10^{-11}$
6	$8.00x10^{-21}$	$8.67x10^{-21}$	$1.65x10^{-21}$	$8.67x10^{-09}$	$4.86x10^{-08}$	$2.42x10^{-09}$	$2.46x10^{-11}$
7	$7.65x10^{-18}$	$1.59x10^{-05}$	$1.32x10^{-15}$	$1.10x10^{-11}$	$6.25x10^{-11}$	$2.83x10^{-09}$	$6.99x10^{-03}$
8	$3.48x10^{-17}$	$2.75x10^{-05}$	$1.23x10^{-04}$	$2.70x10^{-07}$	$9.79x10^{-07}$	$8.46x10^{-06}$	$2.46x10^{-11}$
9	$1.09x10^{-15}$	$8.84x10^{-13}$	$3.23x10^{-03}$	$1.16x10^{-05}$	$2.94x10^{-06}$	$3.57x10^{-04}$	$2.46x10^{-11}$
10	$5.17x10^{-18}$	$2.88x10^{-05}$	$7.42x10^{-11}$	$1.50x10^{-09}$	$1.41x10^{-11}$	$2.85x10^{-08}$	$2.46x10^{-11}$
11	$1.33x10^{-14}$	$5.11x10^{-12}$	NS	$6.44x10^{-03}$	$1.36x10^{-03}$	NS	$2.71x10^{-10}$
12	$1.18x10^{-14}$	2.08x10-^{15}	$7.85x10^{-10}$	$5.90x10^{-14}$	NS	$5.44x10^{-22}$	$1.17x10^{-10}$
13	$2.73x10^{-12}$	$1.00x10^{-10}$	NS	$1.47x10^{-04}$	$7.26x10^{-05}$	$2.71x10^{-06}$	$2.87x10^{-07}$
14	$4.07x10^{-12}$	$6.14x10^{-11}$	$4.32x10^{-05}$	$6.01x10^{-05}$	$6.16x10^{-07}$	$1.28x10^{-15}$	$1.00x10^{-08}$
15	$5.37x10^{-10}$	$3.91x10^{-07}$	NS	$4.22x10^{-04}$	$2.42x10^{-04}$	NS	$3.38x10^{-06}$
16	$6.84x10^{-08}$	$1.97x10^{-04}$	NS	$2.85x10^{-04}$	$5.23x10^{-04}$	NS	$2.25x10^{-07}$
17	$2.14x10^{-08}$	$9.04x10^{-05}$	NS	$3.01x10^{-06}$	$1.36x10^{-05}$	$2.97x10^{-04}$	$4.53x10^{-09}$
18	$1.99x10^{-07}$	NS	NS	$8.14x10^{-03}$	$5.85x10^{-04}$	$2.88x10^{-04}$	$1.45x10^{-06}$
19	$3.74x10^{-07}$	$1.95x10^{-02}$	$2.83x10^{-03}$	$2.15x10^{-08}$	6.11x10-^{07}	$1.81x10^{-07}$	$3.12x10^{-05}$
20	$3.78x10^{-06}$	$2.67x10^{-02}$	NS	$6.57x10^{-05}$	$6.33x10^{-05}$	$6.96x10^{-04}$	$2.11x10^{-05}$
21	$7.37x10^{-06}$	$1.94x10^{-02}$	NS	$7.46x10^{-06}$	$3.69x10^{-06}$	$2.76x10^{-05}$	$1.48x10^{-04}$
22	$1.78x10^{-05}$	$5.85x10^{-04}$	NS	$7.15x10^{-04}$	$2.26x10^{-03}$	$2.43x10^{-05}$	$2.66x10^{-02}$
23	$8.21x10^{-06}$	NS	NS	$1.25x10^{-04}$	$1.11x10^{-02}$	$4.87x10^{-03}$	$5.84x10^{-06}$
24	$3.11x10^{-05}$	$9.71x10^{-04}$	NS	$1.19x10^{-03}$	$8.48x10^{-04}$	$1.13x10^{-03}$	$5.74x10^{-04}$
25	$3.56x10^{-05}$	$2.84x10^{-05}$	NS	NS	NS	$2.06x10^{-02}$	$5.74x10^{-03}$
26	$4.53x10^{-05}$	$1.02x10^{-02}$	NS	$6.63x10^{-06}$	$8.32x10^{-07}$	$2.22x10^{-07}$	NS
27	$7.20x10^{-05}$	NS	NS	$2.89x10^{-09}$	$1.88x10^{-07}$	NS	$8.14x10^{-03}$
28	$1.29x10^{-04}$	$7.41x10^{-05}$	$3.54x10^{-02}$	NS	NS	$2.84x10^{-02}$	$6.69x10^{-03}$
29	$2.19x10^{-04}$	$2.82x10^{-03}$	NS	$5.03x10^{-03}$	$2.83x10^{-02}$	NS	$3.54x10^{-02}$
30	$3.03x10^{-03}$	NS	NS	$6.46x10^{-06}$	$7.28x10^{-06}$	$6.09x10^{-04}$	$3.97x10^{-03}$
31	$3.02x10^{-02}$	$4.80x10^{-03}$	NS	$1.76x10^{-02}$	$2.55x10^{-06}$	NS	$4.04x10^{-03}$

*NS, no signal was detected.
